# *TP53* Gene 72 Arg/Pro (rs1042522) single nucleotide polymorphism increases the risk and the severity of chronic lymphocytic leukemia

**DOI:** 10.3389/fonc.2023.1272876

**Published:** 2023-10-16

**Authors:** Asma Ounalli, Imen Moumni, Amal Mechaal, Aya Chakroun, Mbarka Barmat, Rim El Elj Rhim, Samia Menif, Ines Safra

**Affiliations:** ^1^ Laboratory of Molecular and Cellular Hematology, Pasteur Institute of Tunis, University of Tunis El Manar, Tunis, Tunisia; ^2^ Faculty of Mathematics, Physics and Natural Sciences of Tunis, University of Tunis El Manar, Tunis, Tunisia; ^3^ Department of Hematopoietic Biology and Malignancy, University of Texas MD Anderson Cancer Center, Houston, TX, United States; ^4^ Laboratory of Hematology, Rabta Hospital, Tunis, Tunisia

**Keywords:** chronic lymphocytic leukemia, *TP53* gene, polymorphism 72, hematological parameters, clinical biomarkers

## Abstract

**Background:**

Genetic variations in *TP53* gene are known to be important in chronic lymphocytic leukemia (CLL) and may cause its inactivation which is associated with an aggressive form of the disease. Single nucleotide polymorphism (rs1042522:G>C) in *TP53* gene at codon 72 encodes for arginine (Arg) or proline (Pro) variant which results in amino acid substitution affecting the apoptotic potential of *TP53* protein. The aim of this study was to assess the correlation between *TP53* codon 72 polymorphism and risk susceptibility as well as severity of CLL among Tunisian patients.

**Materials and methods:**

A case-control study was conducted in Tunisia from February 2019 to November 2021, 160 *de novo* CLL patients and 160 healthy volunteers matched in age and gender were involved. DNA was extracted from peripheral blood mononuclear cells and the rs1042522 was analyzed using PCR-RFLP.

**Results:**

Pro variant was associated with higher susceptibility to CLL than Arg variant (p= 0.023). A significant association was found between Pro variant and prognostic classification of Binet stage C (p= 0.001), low hemoglobin level (p= 0.003) and low platelet count (p= 0.016).

**Conclusion:**

We suggest that Pro variant may increase the risk of developing CLL in our population and could be associated with the severity of the disease.

## 1 Introduction

*TP53* is a critical tumor suppressor protein that plays a crucial role in maintaining genomic stability and preventing the development of malignant tumors (1). The *TP53* gene contains 11 exons and is located on the short arm of chromosome 17 (17p13.1) (2). This gene, encodes a 53 kDa protein, plays an important regulatory role in apoptosis, tumor cell proliferation, division, differentiation, migration, maintenance of stem cell characteristics and cell cycle arrest (3, 4).

*TP53* protein contains three functional domains including a transactivation and proline rich domain, a central DNA-binding domain (DBD), and an oligomerization domain (5). The majority of *TP53* missense mutations are located in the central DBD region of *TP53* gene (6).

*TP53* genetic variations are found in over half of human cancers, thus is known as the most commonly mutated gene in human cancers. Different from many other tumor suppressor genes which are generally a deletion or substitution in cancer cells, mutations in *TP53* gene are predominantly missense mutations that give rise to a single amino acid substitution in the full-length mutant protein, plus this protein interacts with several proteins coordinating multiple pathways and favor the activities of oncogenes (7).

*TP53* gene mutations are involved in the pathogenesis of various hematological malignancies, including leukemias and lymphomas (8). They are common in acute myeloid leukemia (AML) (9), in chronic lymphocytic leukemia (CLL) (2), in diffuse large B-cell lymphoma (DLBCL) (10) and in follicular lymphoma (11). Most of *TP53* mutations are associated with chemotherapy resistance and disease relapse, as well as decreased overall survival (12).

In CLL, *TP53* mutations have been found in about 4–37% of patients (13), some are associated with poor prognosis (14).

CLL is the most prevalent leukemia observed in adults, with an incidence rate of 4-6 per 100 000 people annually in USA and in Europe (15). In Tunisia, unfortunately, only a few data are available concerning the epidemiological profile of leukemia. The statistics provided by our laboratory on CLL show that the frequency of this disease is about 105 new cases per year. Total number of leukemia in adults is about 235 new cases per year.

CLL is considered as a category of chronic leukemia with increasing monoclonal B lymphocytes in the bone marrow, peripheral blood and secondary lymphoid tissues (16).

The diagnosis of this pathology is based on the presence of ≥ 5×10^9^ B-lymphocytes/L in the peripheral blood for a duration higher than 3 months. In CLL, cells expression of CD19, CD5, CD20, CD23, kappa and lambda is usually sufficient to establish the diagnosis (17, 18).

The main treatment used for CLL is chemotherapy, monoclonal antibodies and stem cells transplantation, which depends on: the age of patient (< 65 years *vs* ≥ 65 years), clinical status fit or unfit, the Cumulative Illness Rating Scale (CIRS) score, the existence or absence of a 17p deletion and/or the presence of *TP53* mutations. Some patients with CLL can live for many years without the need for treatment (13).

Nowadays, targeted therapy has drastically changed the landscape of hematological disorders including CLL. In fact, it improves outcomes and survival rates, reduces chemotherapy side effects, and enables personalized medicine based on individual genetic and molecular profiles for more effective treatment. Several efforts have been made to develop new strategies that exploit anti-tumor immune responses against *TP53* pathway defects for cancer treatment (19).

The Binet clinical staging system, create prognostic and treatment indication by using results of physical examination and blood counts (**Table 1**) (13, 20). There are several prognostic factors that can impact clinical course starting from indolent to highly progressive disease.

**Table 1:** Binet staging system for CLL.

Binet Stage		
**Stage A**	Hb > 10g/dL, platelets > 100×10^9^/L and <2 lymph node areas involved (*)	Low risk 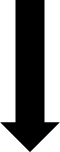 High risk
**Stage B**	Hb > 10g/dL, platelets > 100×10^9^/L and ≥3 lymph node areas involved (*)	
**Stage C**	Hb < 10g/dL or platelets < 100×10^9^/L	

(*) Lymph node areas: 1. Head and neck (uni- or bilateral), 2. Axillar (uni- or bilateral), 3. Inguinal (uni- or bilateral), 4. Splenomegaly, 5. Hepatomegaly.

According to the International Workshop on Chronic Lymphocytic Leukemia (IWCLL) guidelines, criteria of diagnosis of CLL active disease are based on the development of anemia and/or thrombocytopenia, massive or progressive or symptomatic splenomegaly, massive nodes or progressive or symptomatic lymphadenopathy, lymphocyte doubling time (LDT) less than 6 months, autoimmune complications and another disease related symptomes (13).

Non-mutated heavy-chain variable-region genes (IGHVs) and high level of CD38 (≥ 30%) are associated with more aggressive disease compared to those carrying mutated IGHVs and CD38 (< 30%) (21, 22). Other prognostic factors such as the presence of certain comorbidities, blood B cell count, hemoglobin level, and platelet count are also associated with CLL severity (23).

Genetic abnormalities, such as deletions of chromosome 17p and mutations in the *TP53* gene, are important prognostic factors in CLL, as they are associated with resistance to therapy and reduced survival (24).

Several polymorphisms have been detected in *TP53* gene, both in coding and non-coding regions (25). A single-nucleotide variation in *TP53* gene which replaces Arginine (Arg) by Proline (Pro) at codon 72 (rs1042522:G>C), may have an effect on apoptotic functions of *TP53* protein. Arg allele increases ability of *TP53* to locate to mitochondria and induce cellular death, whereas Pro allele owns a lower apoptotic potential and an increased cellular arrest in G0/G1 phases of the cell cycle (26). This polymorphism is reportedly associated with a susceptibility to most cancers. The distribution of the three genotypes (Arg/Arg, Arg/Pro and Pro/Pro) largely relies on the ethnic composition of the studied population.

To the best of our knowledge this is the first study with large sample size conducted in Tunisia to investigate the association between *TP53* Arg72Pro polymorphism and risk of CLL and correlate with clinical and hematological parameters.

## 2 Patients and methods

### 2.1 Study population

Peripheral blood (PB) samples were collected from 160 patients with CLL and 160 age- and sex-matched healthy controls. The samples were then sent to the Laboratory of Molecular and Cellular Hematology at Pasteur Institute of Tunis, Tunisia. Peripheral blood (PB) samples were collected from patients who gave their informed consent and healthy donors between February 2019 and November 2021. This study was approved by the Ethics Committee of Pasteur Institute of Tunis. Clinical data of 49 CLL patients were obtained from their medical records, including sex, age, and a complete blood count. Diagnosis of CLL was based on clinical characteristics, peripheral blood count, morphology, and immunophenotype, with a minimum of 5,000 B lymphocytes per microliter of blood for more than 3 months. CLL cell immunophenotyping was evaluated by flow cytometry, characterized by co-expression of CD5, CD19 and CD23, and low expression of CD20 and kappa or lambda immunoglobulin light chains. All patients who carried Matutes score ≥4 were included in this study (non-CLL cases score below 4) (27). Prognosis of the disease at diagnosis was determined using Binet staging system. CD38 expression levels were analysed. Treatment of CLL was based on the IWCLL criteria (28).

### 2.2 Immunophenotyping by flow cytometry

The CLL diagnosis is based on the immunophenotyping by flow cytometry. This method allows the identification and characterization of malignant B lymphocytes that are characteristic of CLL. To perform this analysis, fresh peripheral blood (PB) samples were stained using a panel of antibodies, including CD45-APC-H7, CD5-APC, CD19-PE-CY7, CD20-PerCP-CY5, CD23-PE, CD22-PerCP-Cy5, IgM-FITC, Kappa-FITC, and Lambda-PE (@BD Biosciences). Matutes scoring system ≥ 4 confirms the CLL diagnosis.

To detect the CD38 expression, CD38-APC-Cy7 (BD Biosciences) was used; a cut-off point of 30% was used to define positivity for CD38.

### 2.3 DNA extraction and genotyping

Blood samples were collected from all patients in EDTA tube (Ethylene Diamine Tetra Acetic Acid) and genomic DNA was extracted from PB mononuclear cells by *salting out* method. *TP53* (NG_017013.2) codon 72 polymorphism was detected using the polymerase chain reaction (PCR)-restriction length polymorphism (RFLP) method. The amplification of a 199 bp fragment containing *TP53* codon 72 polymorphism (G>C) was performed using a forward primer (5’-TTGCCGTCCCAAGCAATGGATGA-3’) and a reverse primer (5’-TCTGGGAAGGGACAGAAGATGAC-3’). The PCR reaction was performed using 2.5 µL of 10X buffer, 0.2 mM dNTP, 2 mM of MgCl2, 0.04 U of Taq polymerase (Applied Biosystems) and 0.2 µM of each forward and reverse exon 4 primers. The thermal cycling conditions were initial denaturation at 95°C for 5 min, followed by 35 cycles of denaturation at 95°C for 30 sec, annealing at 64°C for 1 min, extension at 72°C for 1 min and a final extension for 7 min at 72°C. PCR products were then digested with *BstUI* restriction enzyme (New England Biolabs). PCR products were examined for size and purity using electrophoresis on a 3% agarose gel and visualized under ultraviolet light. The 199 bp fragment remained intact in the presence of the C (Pro) allele. In contrast, it was digested into two fragments of 113 bp and 86 bp in the presence of the G allele (Arg).

### 2.4 Statistical analysis

Patients’ data were collected from patients’ medical files and analyzed using the statistical package for social sciences (SPSS for Windows version 23.0, IBM SPSS Inc., USA) and GraphPad Prism (version 8.0.1). Quantitative variables were shown as mean values ± standard deviation (SD) or median (minimum–maximum) as appropriate. Frequencies of rs1042522 genotypes in patients with CLL and control group were compared using Chi-Square test. Logistic regression was used for calculation of odds ratio (OR) with confidence interval (CI 95%) for risk estimation. The analysis of this polymorphism according to clinico-biological data (sex, age, white blood cell count, red blood cell count, platelets count, hemoglobin level, PNN, lymphocytes, monocytes and the prognostic markers) was compared between the three groups of genotypes: Pro/Pro, Arg/Arg and Arg/Pro using ANOVA statistic test. Statistical differences were considered significant with p< 0.05.

## 3 Results

### 3.1 Study population

This study included a total of 160 patients diagnosed with CLL. The median age of the participants was 67 years with mean of 66.12 ± 12.85 years, ranging from 26 to 88 years. Among the studied cohort, 65% of the patients were males (104 vs 56), resulting in a male: female ratio of 1.85. According to the Binet staging system, the 49 patients with available data were classified as follows: 20 (40.81%) in stage A, 18 (36.73%) in stage B, and 11 (22.44%) in stage C. The medians of white blood cell, red blood cell, platelets and hemoglobin,counts were respectively 32×10^9^/L [10.30 - 246×10^9^/L], 3955×10^9^/L [1430 - 4800×10^9^/L], 200×10^9^/L [29 - 410×10^9^/L] and 120g/L [50 – 160g/L]. When assessing the Matutes score, 22 (44.89%) of CLL patients had a score equal to 4, while 27 (55.10%) had a score equal to 5. Additionally, 28 (57.14%) patients received treatment for CLL, while 21 (42.85%) did not receive treatment. The flow cytometric analysis revealed a CD38 positive expression in 10 (15.62%) patients out of 64.

### 3.2 Distribution of rs1042522

**Figure 1:**
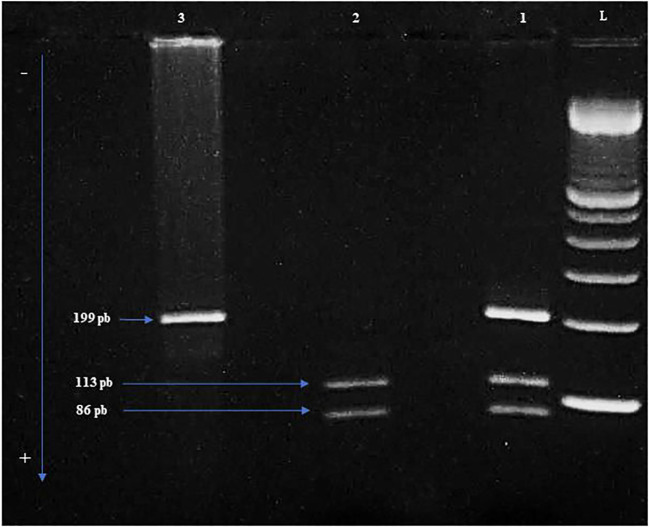
The different genotypes are shown in the electrophoresis gel of Polymerase Chain Reaction-Restriction Fragment Length Polymorphism (PCR-RFLP). L: Ladder of 100 bp. 1: Heterozygous genotype Arg/Pro. 2: Homozygous genotype Arg/Arg. 3: Homozygous genotype Pro/Pro. Arg: Arginine. Pro: Proline.

Our results show the presence of three genotypes of the rs1042522 (**Figure 1**). Genotype frequencies were determined in the CLL patients and the control groups. Arg/Pro was the most frequent genotype in patients with CLL (48.75%), followed by Arg/Arg (42.50%) and Pro/Pro (8.75%). Whereas in healthy control group Arg/Arg was the most frequent genotype (56.25%), followed by Arg/Pro (38.12%) and Pro/Pro (5.62%). The difference between the genotypes of CLL patients and control group was statistically significant (P= 0.044). In CLL patients the frequencies of the Arg and Pro alleles were 214 (66.87%) and 106 (33.12%) respectively, while in the control group, they were 241 (75.3%) and 79 (24.6%), respectively. Notably, the Pro allele was found at a higher frequency in CLL patients compared to the control group (P= 0.023) (**Table 2**).

**Table 2:** Comparison of genotypic and allelic frequencies of the rs1042522 in the CLL patients group versus the control group.

Allele/Genotype	CLL group N=160 (%)	Control group N=160 (%)	Odds ratio (95% CI)	P value
**Arg/Arg Genotype** **Arg/Pro Genotype** **Pro/Pro Genotype**	68 (42.50%) 78 (48.75%) 14 (8.75%)	90 (56.25%) 61 (38.12%) 9 (5.62%)	0.61 (0.25-1.49)	**0.044**
**Arg/Arg Genotype** **Arg/Pro+Pro/Pro Genotypes**	68 (42.57%) 92 (57.50%)	90 (56.25%) 70 (43.75%)	0.57 (0.36-0.89)	**0.018**
**Arg Allele** **Pro Allele**	214 (66.87%) 106 (33.12%)	241 (75.31%) 79 (24.68%)	0.66 (0.46-0.93)	**0.023**

The bold values indicates that they are < 0.05.

### 3.3 Rs1042522 and hematological parameters

**Figure 2:**
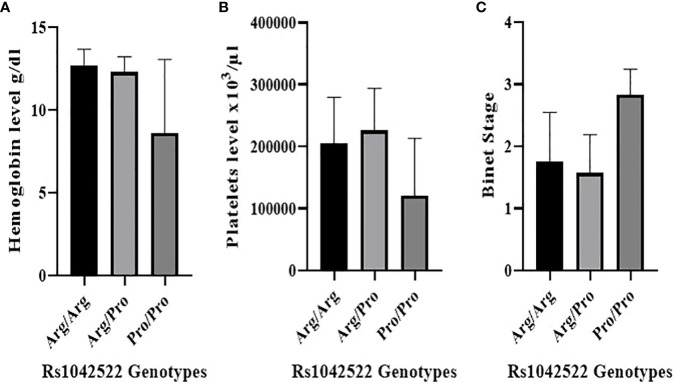
Correlation between rs1042522 genotypes and hematological parameters and Binet Staging system. **(A)** Correlation between rs1042522 genotypes and hemoglobin level. **(B)** Correlation between rs1042522 genotypes and platelets level. **(C)** Correlation between rs1042522 genotypes and Binet stage (1: Stage A, 2: Stage B, 3: Stage C).

The rs1042522 was significantly associated with hemoglobin levels (**Figure 2A**) and platelet counts (**Figure 2B**). Individuals with the Pro homozygous genotype displayed lower hemoglobin levels and platelet counts compared to those with other genotypes (Arg/Arg and Arg/Pro), with p values of 0.003 and 0.016, respectively (**Table 3**).

**Table 3:** Association between the rs1042522 genotypes and hematological parameters in 49 CLL patients.

Parameters	Genotypes			P value
	Arg/Arg	Arg/Pro	Pro/Pro	
	Mean SD	Mean SD	Mean SD	
**WBC (×10^9^/L)**	50.49 41.82	36.77 28.13	91.44 88	0.073
**RBC (×10^9^/L)**	3.81 1.03	4.10 0.42	3.09 1.16	0.194
**Platelets (×10^9^/L)**	205.06 74.56	225.77 68.15	120.66 92.5	**0.016**
**Hemoglobin (g/L)**	12.67 2.39	12.30 1.90	8.61 4.24	**0.003**
**Neutrophils (×10^9^/L)**	6.06 3.23	9.31 8.77	5.53 1.60	0.342
**Lymphocytes (×10^9^/L)**	54.65 80.69	36.33 47.64	88.78 89.95	0.353
**Monocytes (×10^9^/L)**	2.50 3.83	3.21 5.87	2.18 1.47	0.918
**LDH**	358.7 291.17	353.5 147.52	375.66 375.42	0.547
**Beta-2 microglobulin (mg/L)**	4.17 1.88	4.14 2.49	3.30 0.50	0.931

LDH, Lactate dehydrogenase; SD, Standard Deviation.

The bold values indicates that they are < 0.05.

Regarding other blood parameters, no statistically significant differences were observed for total white blood cell count, red blood cell count, neutrophils, lymphocytes, monocytes, LDH, or beta-2-microglobulin concerning the rs1042522 genotype (**Table 3**). However, the results did indicate elevated lymphocyte and white blood cell counts in patients with the Pro/Pro genotype compared to those with other genotypes.

### 3.4 Rs1042522 and biological prognostic markers

The correlation between rs1042522 genotypes and clinical Binet stage revealed that Stage A was associated with Arg/Arg and Arg/Pro genotypes, while Stage C showed a strong association with the Pro/Pro genotype (P value= 0.001) (**Figure 2C**) (**Table 4**).

**Table 4:** Association between the rs1042522 and CLL prognostic markers in 49 CLL patients.

Prognostic Marker	Genotypes			
	Arg/Arg	Arg/Pro	Pro/Pro	P value
**No. Of cases**	68/160 (42.5%)	78/160 (48.75%)	14/160 (8.75%)	
**Mean Age ± SD**	65.33 ± 14.45	66 ± 11.89	69.67 ± 9.93	0.768
**Gender** **Male** **Female**	47/104 (45.19%) 21/56 (37.5%)	47/104 (45.19%) 31/56 (55.35%)	10/104 (9.61%) 4/56 (7.14%)	0.106
**Binet stage** **A** **B** **C**	11/24 (45.83%) 8/24 (33.33%) 5/24 (20.83%)	9/19 (47.36%) 9/19 (47.36%) 1/19 (5.26%)	0/6 (0%) 1/6 (16.66%) 5/6 (83.33%)	**0.001**
**Matutes Score** **4** **5**	10/22 (45.45%) 14/27 (51.85%)	9/22 (40.90%) 10/27 (37.03%)	3/22 (13.63%) 3/27 (11.11%)	0.563
**CD38 expression** **<30%** **>30%**	18/54 (33.33%) 8/10 (80%)	34/54 (62.96%) 2/10 (20%)	2/54 (3.70%) 0/10 (0%)	0.210
**Treatment** **Treated Patient** **No treated Patient**	12/28 (42.85%) 12/21 (57.14%)	11/28 (39.28%) 8/21 (38.09%)	5/28 (17.85%) 1/21 (4.76%)	0.350
**Complications** **Presence** **Absence**	15/28 (53.57%) 9/20 (45%)	9/28 (32.14.1%) 10/20 (50%)	4/28 (14.28.6%) 1/20 (5%)	0.221

The bold values indicates that they are < 0.05.

In the current study, no significant associations were found between gender, median age at diagnosis, Matutes score, biological prognostic marker (CD38 expression), treatment, complications caused by CLL, and the rs1042522 genotypes (**Table 4**).

## 4 Discussion

This is the first case-control study to describe the implication of the *TP53* polymorphism rs1042522 on the risk and the severity of CLL in Tunisian population. The study was conducted to identify CLL patients at risk of severe progression in order to better manage their disease.

CLL is a common malignancy tumor of the hematopoietic system, seriously threatening human health. It is characterized by progressive accumulation of monoclonal and functionally incompetent lymphocytes (29, 30). A variety of factors have been implicated in the occurrence of CLL, including race factors, environmental factors, and family factors (individuals with a family history of leukemia or CLL have a higher risk of CLL) (31). The causes of CLL are not fully understood, and many factors that may contribute to the development of the disease have not been thoroughly studied.

Studies have shown that single nucleotide polymorphisms (SNPs), including those in the *TP53* gene, are associated with an increased risk of CLL (32).

The rs1042522 polymorphism at codon 72 of the *TP53* gene, which encodes either an Arg or Pro amino acid, may alter the biochemical and biological function of *TP53 in vivo*. The Arg variant appears to induce a stronger apoptosis response than the Pro variant (16). On the other hand, CLL patients with the Pro/Pro genotype are considered to be at an increased risk of developing other *TP53* mutations (33).

The clinical effects of this alteration were investigated in several studies. The Pro variant has been shown to be associated with an increased risk of thyroid (34), prostate (35), osteosarcoma (36) and melanoma (37). However, no significant association has been found between the Pro variant and glioma (38, 39) or ovarian cancers (40, 41).

The distribution of rs1042522 polymorphism in our study showed that the Pro/Pro genotype was more frequent in patients with CLL than in the control group. This result is consistent with those published by Basabaeen et al. (42) in the Sudanese population where the frequency of the Pro/Pro genotype was 24.5% in CLL patients and 7.5% in the control group. However, our findings are discordant with those of Kochethu et al. (43) who found that the frequency of the Pro/Pro genotype was 11% in CLL patients and 13% in the control group in the English population.

Many studies showed that rs1042522 frequency differs among populations from various regions in the world. For example, Pro/Pro genotype is represented in about 21.3% of the Chinese population (44). However, in our study, it is present in just 8.75%. This discrepancy may be due to racial differences and variable geographical distributions.

Our results showed the presence of significant association between Pro variant and risk of CLL. We therefore suggest a protective effect of Arg variant on CLL patients.

A previous study conducted in Sudan has reported an association between Arg/Pro genotype and risk of both CLL and acute lymphoblastic leukemia (ALL) (45). Also, a Moroccan study has detected a protective effect of Arg/Arg and the Arg/Pro genotypes (46).

In accordance with Usenova et al. (47), Yang et al. (48) and Dong et al. (44), our study demonstrated no association between rs1042522 genotypes and gender (P=0.106).

In the present study, the mean age of CLL patients was 66.12 ± 12.85 years. Our results showed the absence of significant association between rs1042522 and patients age which is in line with previous studies done by Stomiński et al. (49) and Lahiri et al. (50). Depending on Binet stage system, 40.81% of our patients were presented in Binet stage A, followed by 36.73% at stage B and the rest 22.44% at stage C. Our data demonstrate the presence of a significant association between rs1042522 and Binet stages of our patients. In other words, most of our advanced stage patients (stage C) have the homozygous genotype Pro/Pro (P= 0.001). This result is in accordance with the study of Cabero-Beccera et al. (51). While Sturm et al. (52) reported the absence of any association with Binet stage.

Regarding the expression of CD38, 15.62% of CLL patients were positive, and no association was found with rs1042522 (P= 0.210). This is supported by the previous studies of Sturm et al. (52), Lahiri et al. (50) and Dong et al. (44). However, it is in contrast with a study by Kochethu et al. (43), where authors indicated the presence of a weak association between the homozygous Arg genotype and CD38 negativity (P= 0.049). They found that this genotype was associated with an increased susceptibility to CLL and CD38 negativity but did not appear to influence other biological behavior or clinical response.

Findings of associations between rs1042522 genotype and hematological parameters in CLL in previous reports are contradictory. In the present study, we found that Pro/Pro genotype is associated with hemoglobin level (P= 0.003) and platelet count (P=0.016). In fact, the level of these two hematological parameters seems to be significantly lower for homozygous Pro/Pro genotype than Arg/Arg and Arg/Pro genotypes. Furthermore, the results showed increased lymphocytes and white blood cells for Pro/Pro genotype compared to those with the other genotypes, but without any significant association. However, a recent study showed the absence of any significant association between the different genotypes of this polymorphism and white blood cell count, red blood cell count, platelet count, hemoglobin level, lymphocytes, granulocytes and monocytes (42).

Our study is retrospective and involved a limited number of cases with clinical data. Moreover, the codon 72 polymorphism is not considered a potential prognostic marker. the screening of the entire *TP53* gene and protein expression are required to identify abnormalities associated with CLL prognostic.

In conclusion, this study is the first case-control study to investigate the association between the rs1042522 polymorphism and CLL in the Tunisian population. The results suggest that patients with the Pro/Pro genotype have an increased susceptibility to CLL compared to those with the Arg/Arg or Arg/Pro genotypes. Additionally, the Pro/Pro genotype was associated with lower hemoglobin levels and platelet counts, which are indicative of Binet stage C disease. These findings suggest that the Pro/Pro genotype may be associated with the occurrence and severity of CLL, and may be correlated with other molecular abnormalities of the *TP53* gene.

## Data availability statement

The original contributions presented in the study are included in the article/supplementary material. Further inquiries can be directed to the corresponding author.

## Ethics statement

The studies involving humans were approved by The Ethics Committee of Pasteur Institute of Tunis. The studies were conducted in accordance with the local legislation and institutional requirements. The participants provided their written informed consent to participate in this study.

## Author contributions

AO: Conceptualization, Data curation, Formal Analysis, Investigation, Methodology, Software, Writing – original draft, Writing – review & editing. IM: Formal Analysis, Methodology, Writing – review & editing. AM: Writing – review & editing. AC: Data curation, Writing – review & editing. MB: Data curation, Writing – review & editing. RE: Data curation, Writing – review & editing. SM: Writing – review & editing. IS: Conceptualization, Data curation, Supervision, Validation, Writing – review & editing.

## References

[1] Saeed WH Eissa AA Al-Doski AA. Impact of TP53 gene promoter methylation on chronic lymphocytic leukemia pathogenesis and progression. J Blood Med (2019) 10:399−404. doi: 10.2147/JBM.S221707 31819692 PMC6883927

[2] Moia R Boggione P Mahmoud AM Kodipad AA Adhinaveni R Sagiraju S. Targeting p53 in chronic lymphocytic leukemia. Expert Opin Ther Targets (2020) 24(12):1239−50. doi: 10.1080/14728222.2020.1832465 33016796

[3] Donehower LA Soussi T Korkut A Liu Y Schultz A Cardenas M. Integrated analysis of TP53 gene and pathway alterations in the cancer genome atlas. Cell Rep (2019) 28(5):1370–1384.e5. doi: 10.1016/j.celrep.2019.07.001 31365877 PMC7546539

[4] Marei HE Althani A Afifi N Hasan A Caceci T Pozzoli G. p53 signaling in cancer progression and therapy. Cancer Cell Int (2021) 21(1):703. doi: 10.1186/s12935-021-02396-8 34952583 PMC8709944

[5] Raj N Attardi LD. The transactivation domains of the p53 protein. Cold Spring Harb Perspect Med (2017) 7(1):a026047. doi: 10.1101/cshperspect.a026047 27864306 PMC5204331

[6] Wang H Guo M Wei H Chen Y. Targeting p53 pathways: mechanisms, structures, and advances in therapy. Sig Transduct Target Ther (2023) 8(1):1−35. doi: 10.1038/s41392-023-01347-1 PMC9977964 36859359

[7] Zhu G Pan C Bei JX Li B Liang C Xu Y. Mutant p53 in cancer progression and targeted therapies. Front Oncol (2020) 10:. doi: 10.3389/fonc.2020.595187 PMC7677253 33240819

[8] Stengel A Kern W Haferlach T Meggendorfer M Fasan A Haferlach C. The impact of TP53 mutations and TP53 deletions on survival varies between AML, ALL, MDS and CLL: an analysis of 3307 cases. Leukemia. mars (2017) 31(3):705−11. doi: 10.1038/leu.2016.263 27680515

[9] Granowicz EM Jonas BA. Targeting TP53-mutated acute myeloid leukemia: research and clinical developments. OTT (2022) 15:423−36. doi: 10.2147/OTT.S265637 PMC9037178 35479302

[10] Qin Y Jiang S Liu P Yang J Yang S He X. Characteristics and management of TP53-mutated diffuse large B-cell lymphoma patients. Cancer Manage Res (2020) 12:11515. doi: 10.2147/CMAR.S269624 PMC7666999 33204162

[11] Sorigue M Sancho JM. Current prognostic and predictive factors in follicular lymphoma. Ann Hematol (2018) 97(2):209−27. doi: 10.1007/s00277-017-3154-z 29032510

[12] Molica M Mazzone C Niscola P de Fabritiis P. TP53 mutations in acute myeloid leukemia: still a daunting challenge? Front Oncol (2021) 10:610820. doi: 10.3389/fonc.2020.610820 33628731 PMC7897660

[13] Hallek M Al-Sawaf O. Chronic lymphocytic leukemia: 2022 update on diagnostic and therapeutic procedures. Am J Hematol (2021) 96(12):1679−705. doi: 10.1002/ajh.26367 34625994

[14] Campo E Cymbalista F Ghia P Jäger U Pospisilova S Rosenquist R. TP53 aberrations in chronic lymphocytic leukemia: an overview of the clinical implications of improved diagnostics. Haematologica (2018) 103(12):1956−68. doi: 10.3324/haematol.2018.187583 30442727 PMC6269313

[15] Stauder R Eichhorst B Hamaker ME Kaplanov K Morrison VA Österborg A. Management of chronic lymphocytic leukemia (CLL) in the elderly: a position paper from an international Society of Geriatric Oncology (SIOG) Task Force. Ann Oncol (2017) 28(2):218−27. doi: 10.1093/annonc/mdw547 27803007

[16] Jalilian N Maleki Y Shakiba E Aznab M Rahimi Z Salimi M. p53 p.Pro72Arg (rs1042522) and Mouse Double Minute 2 (MDM2) Single-Nucleotide Polymorphism (SNP) 309 Variants and Their Interaction in Chronic Lymphocytic Leukemia(CLL): A Survey in CLL Patients from Western Iran. Int J Hematol Oncol Stem Cell Res (2021) 15(3):160−9. doi: 10.18502/ijhoscr.v15i3.6846 35082997 PMC8748241

[17] Ginaldi L De Martinis M Matutes E Farahat N Morilla R Catovsky D. Levels of expression of CD19 and CD20 in chronic B cell leukaemias. J Clin Pathol (1998) 51(5):364−9. doi: 10.1136/jcp.51.5.364 9708202 PMC500695

[18] Moreau EJ Matutes E A’Hern RP Morilla AM Morilla RM Owusu-Ankomah KA. Improvement of the chronic lymphocytic leukemia scoring system with the monoclonal antibody SN8 (CD79b). Am J Clin Pathol (1997) 108(4):378−82. doi: 10.1093/ajcp/108.4.378 9322589

[19] Perutelli F Jones R Griggio V Vitale C Coscia M. Immunotherapeutic strategies in chronic lymphocytic leukemia: advances and challenges. Front Oncol (2022) 12:837531. doi: 10.3389/fonc.2022.837531 35265527 PMC8898826

[20] Hallek M. Chronic lymphocytic leukemia: 2020 update on diagnosis, risk stratification and treatment. Am J Hematology. (2019) 94(11):1266−87. doi: 10.1002/ajh.25595 31364186

[21] Damle RN Wasil T Fais F Ghiotto F Valetto A Allen SL. Ig V gene mutation status and CD38 expression as novel prognostic indicators in chronic lymphocytic leukemia. Blood (1999) 94(6):1840−7. doi: 10.1182/blood.V94.6.1840.418k06_1840_1847 10477712

[22] Zenz T Mertens D Küppers R Döhner H Stilgenbauer S. From pathogenesis to treatment of chronic lymphocytic leukaemia. Nat Rev Cancer (2010) 10(1):37−50. doi: 10.1038/nrc2764 19956173

[23] Korubo KI Okite UP Ezeugwu SI. Chronic lymphocytic leukemia: prognostic factors at presentation in a resource-limited center. JCO Global Oncol (2021) 7):56−62. doi: 10.1200/GO.20.00276 PMC8081540 33434067

[24] Chauffaille M de LLF Zalcberg I Barreto WG Bendit I. Detection of somatic TP53 mutations and 17p deletions in patients with chronic lymphocytic leukemia: a review of the current methods. Hematol Transfus Cell Ther (2020) 42(3):261−8. doi: 10.1016/j.htct.2020.05.005 32660851 PMC7417461

[25] Sharma S Sambyal V Guleria K Manjari M Sudan M Uppal MS. TP53 polymorphisms in sporadic north Indian breast cancer patients. Asian pacific journal of cancer prevention. Asian Pacific J Cancer Prevention (2014) 15(16):6871−9. doi: 10.7314/APJCP.2014.15.16.6871 25169539

[26] Lotfi Garavand A Mohammadi M Mohammadzadeh S. Evaluation of TP53 codon 72, P21 codon 31, and MDM2 SNP309 polymorphisms in Iranian patients with acute lymphocytic leukemia. Rep Biochem Mol Biol (2020) 9(1):26−32. doi: 10.29252/rbmb.9.1.26 32821748 PMC7424426

[27] Panda D Tejwani N Mehta A. Chronic lymphocytic leukemia: Current approach to lab diagnosis. J Curr Oncol (2022) 5(1):39. doi: 10.4103/jco.jco_12_22

[28] Hallek M Cheson BD Catovsky D Caligaris-Cappio F Dighiero G Döhner H. iwCLL guidelines for diagnosis, indications for treatment, response assessment, and supportive management of CLL. Blood (2018) 131(25):2745−60. doi: 10.1182/blood-2017-09-806398 29540348

[29] Bird ST Tian F Flowers N Przepiorka D Wang R Jung TH. Idelalisib for treatment of relapsed follicular lymphoma and chronic lymphocytic leukemia: A comparison of treatment outcomes in clinical trial participants vs medicare beneficiaries. JAMA Oncol (2020) 6(2):248−54. doi: 10.1001/jamaoncol.2019.3994 31855259 PMC6990831

[30] Shi K Sun Q Qiao C Zhu H Wang L Wu J. 98% IGHV gene identity is the optimal cutoff to dichotomize the prognosis of Chinese patients with chronic lymphocytic leukemia. Cancer Med (2020) 9(3):999−1007. doi: 10.1002/cam4.2788 31849198 PMC6997101

[31] Kipps TJ Stevenson FK Wu CJ Croce CM Packham G Wierda WG. Chronic lymphocytic leukaemia. Nat Rev Dis Primers. (2017) 3:16096. doi: 10.1038/nrdp.2016.96 28102226 PMC5336551

[32] Zhang WJ Guo SL Yin G Wang GS Wang ZR Dong J. Association between TP53 polymorphisms and chronic lymphocytic leukemia. Eur Rev Med Pharmacol Sci (2020) 24(23):12073−9. doi: 10.26355/eurrev_202012_23996 33336725

[33] Bilous NI Abramenko IV Chumak AA Dyagil IS Martіna ZV. TP53 codon 72 single nucleotide polymorphism in chronic lymphocytic leukemia. Exp Oncol (2014) 36(4):258−61. 25537220

[34] Khan MS Pandith AA Masoodi SR Khan SH Rather TA Andrabi KI. Significant association of TP53 Arg72Pro polymorphism in susceptibility to differentiated thyroid cancer. Cancer Biomarkers. (2015) 15(4):459−65. doi: 10.3233/CBM-150485 25835179 PMC12965092

[35] Lu Y Liu Y Zeng J He Y Peng Q Deng Y. Association of p53 codon 72 polymorphism with prostate cancer: an update meta-analysis. Tumor Biol (2014) 35(5):3997−4005. doi: 10.1007/s13277-014-1657-y 24488627

[36] Huang X Wu F Zhang Z Shao Z. Association between TP53 rs1042522 gene polymorphism and the risk of Malignant bone tumors: a meta-analysis. Bioscience Rep (2019) 39(3):BSR20181832. doi: 10.1042/BSR20181832 PMC6422898 30833364

[37] Geng P Liao Y Ruan Z Liang H. Increased risk of cutaneous melanoma associated with p53 Arg72Pro polymorphism. PloS One (2015) 10(3):e0118112. doi: 10.1371/journal.pone.0118112 25774791 PMC4361629

[38] Liao F Yuan L Zhu J Chen W Zhao Y He J. Association of TP53 rs1042522 C>G polymorphism with glioma risk in chinese children. BioMed Res Int (2022) 2022:e2712808. doi: 10.1155/2022/2712808 PMC9392611 35996546

[39] Nerweyi FFA. The relationship of gliomas with tp53 rs1042522 c > g and gliomas in duhok. J Duhok Univ (2020) 23(2):157−65. doi: 10.26682/sjuod.2020.23.2.17

[40] Alqumber MAA Akhter N Haque S Panda AK Mandal RK. Evaluating the Association between p53 Codon 72 Arg>Pro Polymorphism and Risk of Ovary Cancer: A Meta-Analysis. PloS One (2014) 9(4):e94874. doi: 10.1371/journal.pone.0094874 24747975 PMC3991634

[41] Brenner PK Kapralova MA Khodyrev DS Khokhlova SV Khabas GN Asaturova AV. Association of polymorphic markers of the TP53, MDM2, and CDKN1A genes with the risk of ovarian cancer. Russ J Genet (2022) 58(9):1154−8. doi: 10.1134/S102279542209006X

[42] Mohammed Basabaeen AA Abdelgader EA Babekir EA Abdelrahim SO Eltayeb NH Altayeb OA. TP53 gene 72 arg/pro (rs1042522) single nucleotide polymorphism contribute to increase the risk of B-chronic lymphocytic leukemia in the Sudanese population. Asian Pac J Cancer Prev (2019) 20(5):1579−85. doi: 10.31557/APJCP.2019.20.5.1579 31128065 PMC6857868

[43] Kochethu G Delgado J Pepper C Starczynski J Hooper L Krishnan S. Two germ line polymorphisms of the tumour suppressor gene p53 may influence the biology of chronic lymphocytic leukaemia. Leuk Res (2006) 30(9):1113−8. doi: 10.1016/j.leukres.2005.12.014 16458962

[44] Dong HJ Fang C Wang L Fan L Xu J Wu JZ. TP53 Pro72 allele potentially increases the poor prognostic significance of TP53 mutation in chronic lymphocytic leukemia. Med Oncol (2014) 31(4):908. doi: 10.1007/s12032-014-0908-5 24615009

[45] Ahmed MAM Mirghani LB Ali EW. Genotyping of p53 exon 4 codon 72 in Sudanese patients with lymphoid leukaemias. International J Current Res (2013) 4:3980–3.

[46] Skhoun H Khattab M Belkhayat A Takki Chebihi Z Bakri Y Dakka N. Association of TP53 gene polymorphisms with the risk of acute lymphoblastic leukemia in Moroccan children. Mol Biol Rep (2022) 49(9):8291−300. doi: 10.1007/s11033-022-07643-3 35705773

[47] Usenova A Akhunbaev S Tumanbaev A Lim E. The Relationship between Gene Polymorphisms od the XRCC1 and TP53 with the Gender of Children with Acute Leukemia. Asian Pacific J Cancer Prev (2023) 24(2):613−21. doi: 10.31557/APJCP.2023.24.2.613 PMC10162603 36853312

[48] Yang T Wen Y Li J Yang J Tan T Pan J. Association of the TP53 rs1042522 C>G polymorphism and hepatoblastoma risk in Chinese children. J Cancer (2019) 10(15):3444−9. doi: 10.7150/jca.33063 31293648 PMC6603402

[49] Słomiński B Skrzypkowska M Ryba-Stanisławowska M Myśliwiec M Trzonkowski P. Associations of TP53 codon 72 polymorphism with complications and comorbidities in patients with type 1 diabetes. J Mol Med (2021) 99(5):675−83. doi: 10.1007/s00109-020-02035-1 33495869 PMC8055568

[50] Lahiri O Harris S Packham G Howell M. p53 pathway gene single nucleotide polymorphisms and chronic lymphocytic leukemia. Cancer Genet Cytogenetics (2007) 179(1):36−44. doi: 10.1016/j.cancergencyto.2007.07.013 17981213

[51] Cabero-Becerra M Garcia Vela JA Sanchez-Godoy P Arias-Arias A Piris-Villaespesa M Pérez - Sanz N. Increase in mortality and second neoplasms in chronic lymphocytic leukemia with pro/pro genotype of TP53 codon 72. Blood (2020) 136(Supplement 1):13−4. doi: 10.1182/blood-2020-141768

[52] Sturm I Bosanquet AG Hummel M Dörken B Daniel PT. In B-CLL, the codon 72 polymorphic variants of p53 are not related to drug resistance and disease prognosis. BMC Cancer. (2005) 5(1):105. doi: 10.1186/1471-2407-5-105 16109171 PMC1208864

